# Impact of angiogenesis inhibitor eligibility on the prognosis of patients with non‐small cell lung cancer harboring *EGFR* mutation

**DOI:** 10.1002/cam4.4268

**Published:** 2021-09-29

**Authors:** Hiroaki Kodama, Hirotsugu Kenmotsu, Takanori Kawabata, Akifumi Notsu, Michitoshi Yabe, Naoya Nishioka, Eriko Miyawaki, Taichi Miyawaki, Nobuaki Mamesaya, Haruki Kobayashi, Shota Omori, Kazushige Wakuda, Akira Ono, Tateaki Naito, Haruyasu Murakami, Toshiaki Takahashi

**Affiliations:** ^1^ Division of Thoracic Oncology Shizuoka Cancer Center Shizuoka Japan; ^2^ Department of Biostatistics Clinical Research Center Shizuoka Cancer Center Shizuoka Japan

**Keywords:** angiogenesis inhibitor, epidermal growth factor tyrosine kinase inhibitor, non‐small cell lung cancer, vascular endothelial growth factor

## Abstract

**Background:**

Epidermal growth factor receptor tyrosine kinase inhibitors (EGFR‐TKIs) are currently the primary treatment option for patients with *EGFR*‐mutant non‐small cell lung cancer (NSCLC). However, the effect of EGFR‐TKIs are eventually weakened due to resistance, and there is also a differential efficacy based on EGFR mutation subtypes. The combination of angiogenesis inhibitor (AI) with EGFR‐TKI has shown better efficacy than EGFR‐TKI monotherapy, regardless of the mutation subtypes. Nevertheless, the effect of AI eligibility on overall survival (OS) and progression‐free survival (PFS) remains to be elucidated. Thus, we assessed this impact on patients with NSCLC harboring *EGFR* mutation.

**Methods:**

In this study, the data for 450 patients with *EGFR*‐mutant NSCLC, who were treated with EGFR‐TKI monotherapy, were retrospectively analyzed for AI eligibility. The patients were categorized into AI‐eligible (AI fit) and ineligible groups (AI unfit).

**Results:**

The median PFS of the AI fit group was 12.9 months, compared to 9.6 months in the unfit group (*p* = 0.007), and OS was also significantly longer in the AI fit group (median OS = 33.0 months) compared to that in the unfit group (18.5 months, *p* < 0.001). Multivariate analysis indicated that AI ineligibility was associated with shorter PFS and poor prognosis. Also, in the AI fit group, there was no significant difference in the PFS between *EGFR* L858R mutation and EGFR exon 19 deletion (median PFS = 11.5 months vs. 13.8 months; *p* = 0.17).

**Conclusions:**

From our study, AI eligibility resulted in longer OS and PFS, and also had different effects on patients with EGFR L858R and exon 19 deletion. Since this selection bias may have affected previous clinical trial data on the efficacy of AI combination therapy, their results should be carefully considered henceforth.

## Graphical abstract

We assessed the impact of angiogenesis inhibitor (AI) eligibility on epidermal growth factor receptor mutant non‐small cell lung cancer. AI eligibility was associated with longer progression‐free survival and overall survival. Selection bias may affect previous data on the efficacy of AI combination therapy.

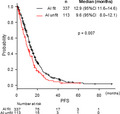

## 1 INTRODUCTION

Lung cancer is one of the major causes of cancer‐related deaths in the world. Although the prognosis remains limited compared to that of other types of cancers, the evolution of molecular targeted therapy has drastically improved the prognosis, especially in driver mutation‐positive non‐small cell lung cancer (NSCLC). Epidermal growth factor receptor (*EGFR*) is one of the most essential oncogenes considered when developing a treatment for *EGFR*‐mutant NSCLC. Treatment with EGFR‐tyrosine kinase inhibitors (TKIs) has enabled significantly prolonged outcomes for patients compared to conventional platinum‐based combination chemotherapy. EGFR‐TKIs became the standard first‐line regimen for patients with NSCLC harboring an *EGFR* mutation. Yet, the benefits of EGFR‐TKIs eventually fade because of acquired resistance to TKI treatment. Additionally, previous EGFR‐TKI monotherapy studies^1^, ^2^, ^3^, ^4^, ^5^ have described a difference in the efficacy of EGFR‐TKI usage between different *EGFR* mutation subtypes and have suggested that tumors with exon 19 deletion (19 del) exhibit a higher sensitivity to EGFR‐TKI treatment compared to those with EGFR an exon 21 point mutation (L858R). Thus, novel treatment regimens for NSCLC harboring *EGFR* mutations are needed to prolong the survival time and reduce the imbalance in the effectiveness between *EGFR* subtypes.

Recently, the EGFR‐TKI plus angiogenesis inhibitors (AIs) combination therapy has demonstrated better efficacy than EGFR‐TKI monotherapy in patients with *EGFR*‐mutant NSCLC. In the recent NEJ026 study, a randomized open‐label phase 3 study, *EGFR* mutation‐positive NSCLC patients who received bevacizumab (a humanized monoclonal antibody to vascular endothelial growth factor [VEGF]) plus erlotinib combination therapy showed significantly prolonged progression‐free survival (PFS) compared with those who received erlotinib monotherapy.^6^ In addition, administering erlotinib plus ramucirumab (a human monoclonal IgG1 antibody that targets VEGFR‐2) shows improved PFS compared with administering erlotinib alone.^7^ Although these randomized clinical trials have demonstrated the improvement of PFS in AI combination therapy, these studies have failed to show a survival benefit. Besides their efficacy, AI combination therapies also show a comparable PFS in tumors with an L858R mutation and exon 19 deletion. Based on these results, AI combination therapies are expected to become a compelling treatment option for patients with NSCLC harboring an *EGFR* mutation, especially the L858R subtype.

However, because of the unique eligibility criteria for AIs, some scientists express concerns about a selection bias in AI combination clinical studies and believe that a better prognosis can be achieved even without the administration of AIs.^8^, ^9^ Moreover, whether the eligibility criteria for AI affect the prognosis of NSCLC patients with activating *EGFR* mutation is also unclear. Thus, in this study, we aimed to assess the prognostic impact of AI eligibility criteria on patients with NSCLC with *EGFR* mutation and compare the effectiveness of EGFR‐TKI monotherapy in patients with an L858R mutation to that in those with exon 19 deletion in the AI‐eligible population.

## 2 METHODS

### 2.1 Study participants

Patients with an EGFR‐mutant (L858R or 19 del) NSCLC, who started first‐line EGFR‐TKI treatment at Shizuoka Cancer Center between 2002 and 2019 were collected retrospectively. We excluded patients whose Eastern Cooperative Oncology Group (ECOG) performance status (PS) was 3 or 4, who had been treated by any AIs, and patients with symptomatic brain metastasis. We defined the enrolled patients as the ALL group. In the ALL group, we defined patients as AI‐ineligible if they met at least one of the following conditions: (1) A history of tumor exposure in the bronchus or of producing bloody sputum; (2) a major vessel infiltration (MVI) by the tumor, diagnosed by a radiologist; (3) a history of cardiovascular disease (CVD), including thrombotic diseases, ischemic heart diseases, or congestive heart failure; (4) treatment by chemoradiotherapy before initiation of EGFR‐TKI monotherapy. Since there were no cases with active peptic ulcer disease before treatment, we did not include active peptic ulcer disease as the classifying condition. Next, we evaluated the effects of first‐line EGFR‐TKI monotherapy in both AI‐eligible (AI fit) and ineligible (AI unfit) groups. We also evaluated the efficacies of EGFR‐TKI monotherapy in patients with exon 19 deletion or L858R mutation in the AI fit and unfit groups. PFS was defined as the time from the start of EGFR‐TKI monotherapy to death, disease progression, or censoring at the last follow‐up examination. A considerable number of cases showed several months or years of a treatment‐free period due to EGFR‐TKI toxicity before disease progression or initiation of sequential treatment. Therefore, patients who started new therapy without confirmation of tumor progression were censored at the time of the latest tumor assessment before the new therapy was initiated. We defined overall survival (OS) as the interval between the initiation of EGFR‐TKI therapy, and death from any cause or censoring at the last follow‐up. *EGFR* mutations in tumor tissues were detected using cobas® *EGFR* mutation test v2 (Roche Molecular Systems), CycleavePCR^™^ Assay (TAKARA, Co., Ltd.), or scorpion arms assay (DxS). Tumor response was evaluated using Response Evaluation Criteria in Solid Tumor criteria version 1.1.^10^

This study was authorized by the institutional review board of Shizuoka Cancer Center.

### 2.2 Statistical analysis

Patient characteristics were compared between the AI fit and unfit groups and patients with L858R and exon 19 deletion, using the Mann–Whitney *U* test for all continuous variables, and Fisher's exact test for the categorical data. Clinical evaluation of PFS and OS was conducted using the Kaplan–Meier method. We used the Log‐rank test to compare the cumulative survival in each group and Cox's proportional hazards analysis for the multivariate analysis. All *p* values were two‐sided, and *p* < 0.05 was considered significant. Statistical analyses were performed using EZR (Saitama Medical Center, Jichi Medical University), which is a converted version that added frequently‐used biostatistical functions to an original R commander (version 1.6‐3).^11^

## 3 RESULTS

### 3.1 Patient characteristics

**FIGURE 1:**
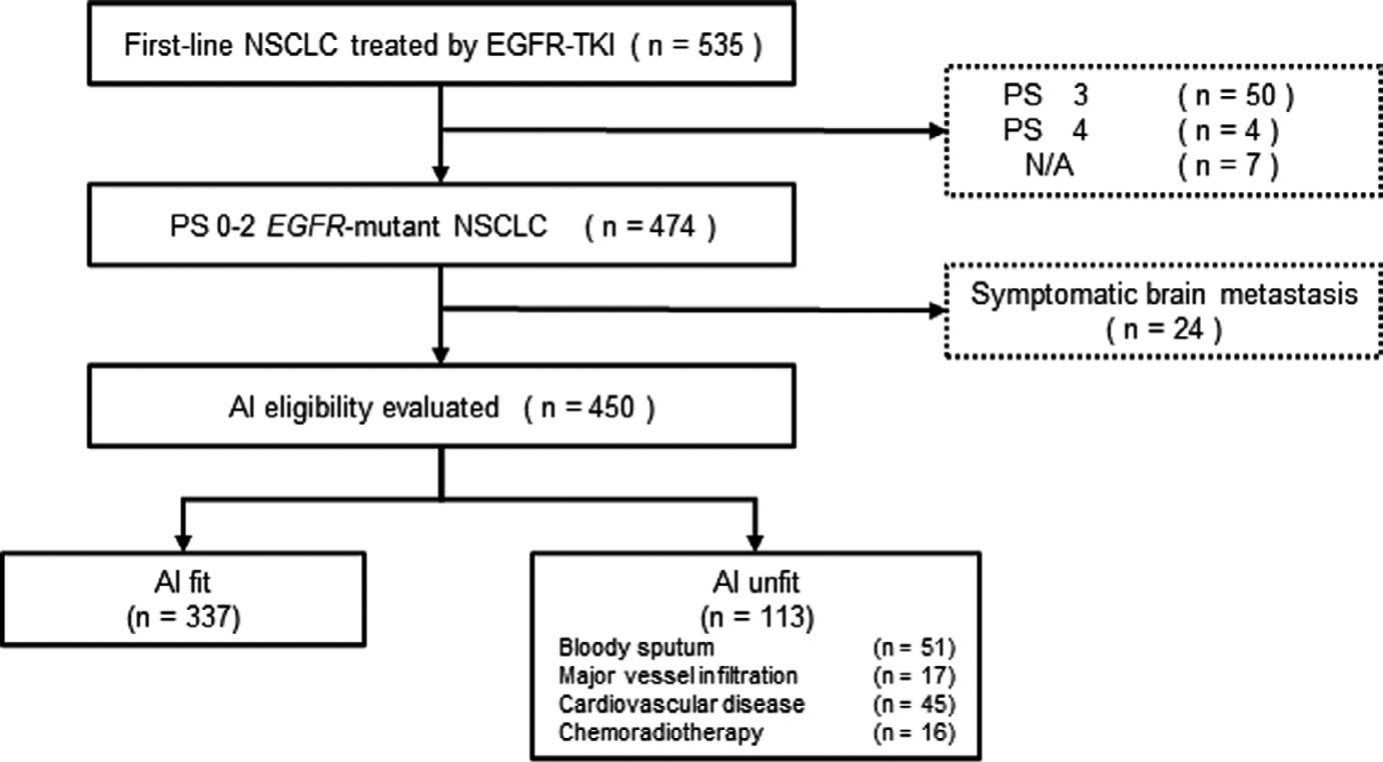
Flow chart of all the patients. AI, angiogenesis inhibitor; EGFR, epidermal growth factor receptor; NSCLC, non‐small cell lung cancer; TKI, tyrosine kinase inhibitor

In total, 535 *EGFR* major mutation‐positive patients had been treated with first‐line EGFR‐TKIs monotherapy during the study term (Figure 1). We excluded 85 patients, including 50 patients with PS 3, four with PS 4, seven without PS data, and 24 with symptomatic brain metastasis. Overall, 450 patients were included in the study (ALL group), and the background information is shown in Table 1.

**TABLE 1:** Characteristics of all patients and those in the AI fit and unfit groups

	Overall	AI fit	AI unfit	*p* value
	(*N* = 450)	(*N* = 337)	(*N* = 113)	
Age, years				0.019
Median	71	72	70	
Range	31–92	34–90	31–92	
<75	314 (69.8)	241 (71.5)	73 (64.6)	0.193
≦75	136 (30.2)	96 (28.5)	40 (35.4)	
Sex, *n* (%)				0.140
Male	157 (34.9)	111 (32.9)	46 (40.7)	
Female	293 (65.1)	226 (67.1)	67 (59.3)	
Stage, *n* (%)				0.017
Relapse after surgery	126 (28.0)	90 (26.7)	36 (31.9)	
IIIB	10 (2.2)	4 (1.2)	6 (5.3)	
IV	314 (69.8)	243 (72.1)	71 (62.8)	
ECOG PS, *n* (%)				0.026
0	128 (28.4)	104 (30.9)	24 (21.2)	
1	249 (55.3)	186 (55.2)	63 (55.8)	
2	73 (16.2)	47 (13.9)	26 (23.0)	
Smoking history, *n* (%)				0.743
Never	256 (56.9)	190 (56.4)	66 (58.4)	
Former/never	194 (43.1)	147 (43.6)	47 (41.6)	
Brain metastasis, *n* (%)				1.000
Yes (asymptomatic)	140 (31.1)	105 (31.2)	35 (31.0)	
No	310 (68.9)	232 (68.8)	78 (69.0)	
*EGFR* subtype, *n* (%)				0.514
L858R	206 (45.8)	151 (44.8)	55 (48.7)	
19 del	244 (54.2)	186 (55.2)	58 (51.3)	
EGFR‐TKI, *n* (%)				0.127
Gefitinib	273 (60.7)	200 (59.3)	73 (64.6)	
Erlotinib	85 (18.9)	59 (17.5)	26 (23.0)	
Afatinib	31 (6.9)	26 (7.7)	5 (4.4)	
Osimertinib	61 (13.6)	52 (15.4)	9 (8.0)	

Abbreviations: 19 del, exon 19 deletion; AI, angiogenesis inhibitor; ECOG PS, Eastern Cooperative Oncology Group performance status; *EGFR*, epidermal growth factor receptor; TKI, tyrosine kinase inhibitor.

This study included 293 female patients (65.1%), and the median age at the initiation of EGFR‐TKI therapy was 71 years (range 31–92). Most patients were diagnosed with stage IV disease (69.8%), and 28.0% relapsed after the surgery. Around 50% of the enrolled patients had a history of smoking, and 45.8% had an L858R mutation. Most patients underwent gefitinib treatment (60.7%), followed by erlotinib (18.9%), osimertinib (13.6%), and afatinib (6.9%). As per the four factors of AI eligibility, 113 patients were defined as AI‐ineligible, of which 51 patients had a history of tumor exposure in the bronchus or of producing bloody sputum, 17 had MVI, 45 had a history of CVD, and 16 had been treated with chemoradiotherapy before starting the EGFR‐TKI treatment. Both groups (AI fit group and AI unfit group) showed similar patient characteristics regarding sex, smoking history, *EGFR* subtype, and first EGFR‐TKI drug. However, there was a significant disproportion in the two groups based on ECOG PS (*p* = 0.03) and age (*p* = 0.019).

### 3.2 Efficacy of EGFR‐TKI in the AI fit and unfit groups

**FIGURE 2:**
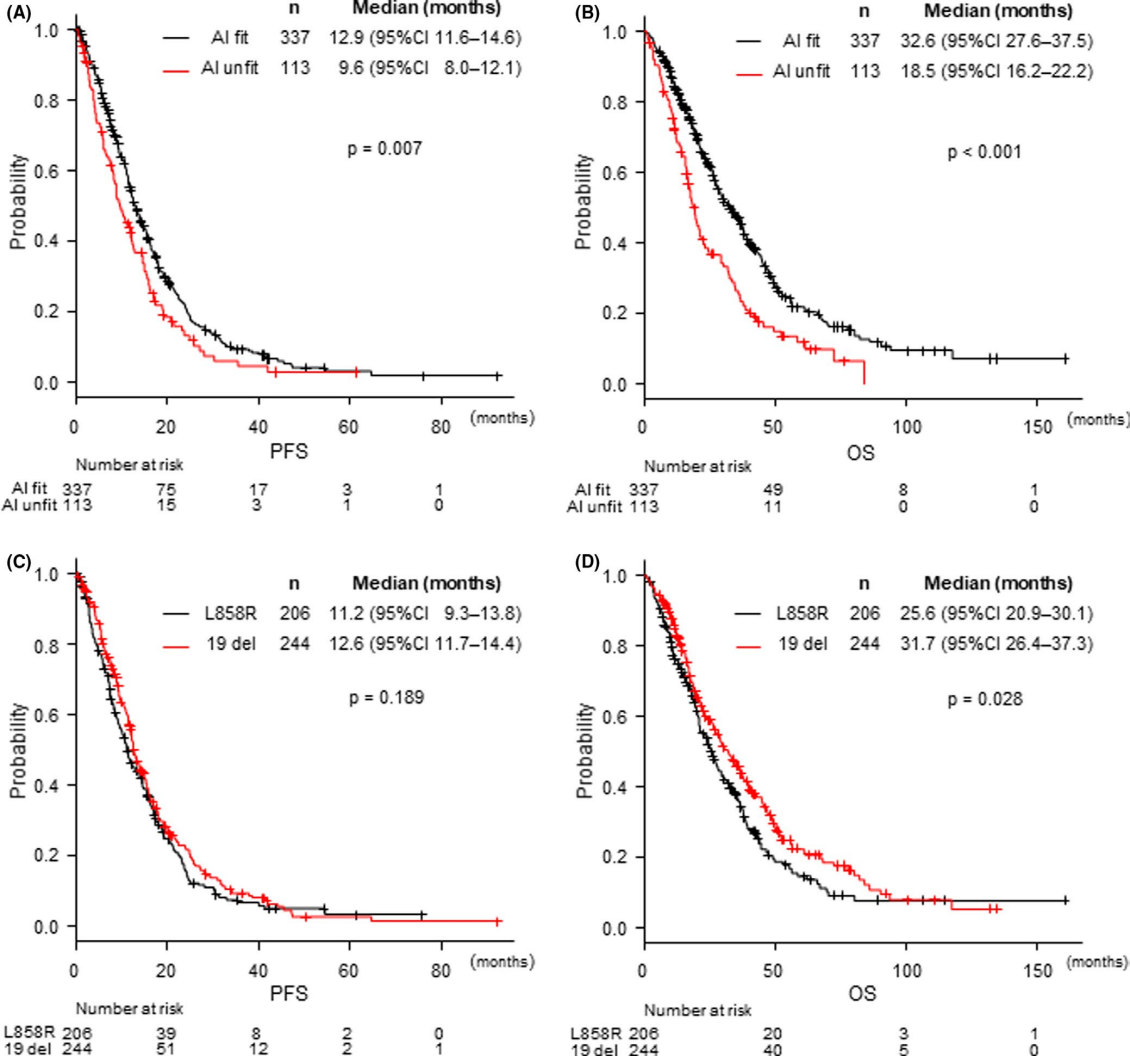
Kaplan–Meier curves for (A) progression‐free survival and (B) overall survival in the AI fit and unfit groups, and (C) progression‐free survival and (D) overall survival in patients with an L858R mutation or an exon 19 deletion. AI, angiogenesis inhibitor; CI, confidence interval; HR, hazard ratio; mOS, median overall survival; mPFS, median progression‐free survival

With a median follow‐up period of 55.8 months (95% CI: 48.2–66.4 months) (Kaplan–Meier estimate), the PFS was significantly better in the AI fit group (median PFS = 12.9 months) than in that the AI unfit group (9.6 months; hazard ratio [HR] = 0.73, 95% confidence interval [CI] = 0.57–0.92; *p* = 0.007; Figure 2A). Multivariate analysis of PFS in the ALL group indicated that AI eligibility (HR = 0.75; 95% CI: 0.59–0.95; *p* = 0.018), stage (HR = 0.57; 95% CI: 0.45–0.72; *p* < 0.001), and PS (HR = 0.56; 95% CI: 0.42–0.75, *p* < 0.001) were significantly associated with PFS (Table 2A). Similarly, the OS of AI fit group (median OS = 32.6 months) was significantly longer than that of the AI unfit group (18.5 months; HR = 0.58; 95% CI: 0.45–0.74; *p* < 0.001; Figure 2B).

**TABLE 2:** Multivariate analysis in the ALL group

	*n*	%	Univariate		Multivariate	
			HR (95% CI)	*p* value	HR (95% CI)	*p* value
(A) PFS						
AI fit/unfit	337/113	74.9/25.1	0.73 (0.57–0.92)	**0.007**	0.75 (0.59–0.95)	**0.018**
Age (<74/≧75)	314/136	69.8/30.2	1.08 (0.86–1.35)	0.519	1.04 (0.83–1.31)	0.731
Sex (male/female)	157/293	34.9/65.1	1.34 (1.08–1.66)	**0.007**	1.22 (0.92–1.61)	0.172
Stage (relapse, IIIB/IV)	136/314	30.2/69.7	0.58 (0.46–0.74)	**<0.001**	0.57 (0.45–0.72)	**<0.001**
PS (0,1/2)	377/73	83.8/16.2	0.59 (0.45–0.79)	**<0.001**	0.56 (0.42–0.75)	**<0.001**
Smoke (former, current/never)	256/194	56.9/43.1	1.29 (1.05–1.59)	**0.015**	1.15 (0.88–1.51)	0.308
*EGFR* (L858R/19 del)	206/244	45.8/54.2	1.15 (0.93–1.41)	0.191	1.18 (0.96–1.45)	0.122
TKI (first‐, second‐generation/osimertinib)	389/61	86.4/13.6	1.32 (0.89–1.96)	0.168	1.52 (1.02–2.27)	**0.040**
(B) OS						
AI fit/unfit	337/113	74.9/25.1	0.58 (0.45–0.74)	**<0.001**	0.60 (0.47–0.77)	**<0.001**
Age (<74/≧75)	314/136	69.8/30.2	0.75 (0.59–0.96)	**0.020**	0.74 (0.58–0.95)	**0.016**
Sex (male/female)	157/293	34.9/65.1	1.51 (1.20–1.89)	**<0.001**	1.39 (1.02–1.90)	**0.038**
Stage (relapse, IIIB/IV)	136/314	30.2/69.7	0.71 (0.55–0.91)	**0.007**	0.71 (0.55–0.92)	**0.009**
PS (0,1/2)	377/73	83.8/16.2	0.51 (0.38–0.69)	**<0.001**	0.49 (0.36–0.66)	**<0.001**
Smoke (former, current/never)	256/194	56.9/43.1	1.34 (1.07–1.68)	**0.010**	1.18 (0.87–1.61)	0.290
*EGFR* (L858R/19 del)	206/244	45.8/54.2	1.28 (1.03–1.60)	**0.029**	1.33 (1.06–1.66)	**0.014**
TKI (first‐, second‐generation/osimertinib)	389/61	86.4/13.6	1.20 (0.68–2.12)	0.537	1.24 (0.70–2.21)	0.455

Abbreviations: 19 del, exon 19 deletion; CI, confidence interval; *EGFR*, epidermal growth factor receptor; first‐, second‐generation, gefitinib, erlotinib, and afatinib; HR, hazard ratio; PFS, progression‐free survival; PS, performance status; TKI, tyrosine kinase inhibitor.

Bold values are *p* <0.05, which is statistically significant.

In the multivariate analysis of the OS, AI eligibility (HR = 0.60, 95% CI: 0.47–0.77, *p* < 0.001), age (HR = 0.74, 95% CI: 0.58–0.95, *p* = 0.016), sex (HR = 1.39, 95% CI: 1.02–1.90, *p* = 0.038), PS (HR = 0.49, 95% CI: 0.36–0.66, *p* < 0.001), stage (HR = 0.71, 95% CI: 0.55–0.92, *p* = 0.009), and *EGFR* subtype (HR = 1.33, 95% CI: 1.06–1.66, *p* = 0.014) were indicated as prognostic factors (Table 2B). Among the four AI eligibility criteria, a history of tumor exposure in the bronchus or producing bloody sputum was related to a shorter PFS (HR = 1.43, 95% CI: 1.04–1.97, *p* = 0.027), and was indicated to be a significant prognostic factor for the OS (HR = 1.61, 95% CI: 1.14–2.28, *p* = 0.007).

### 3.3 Differences in *EGFR* subtypes in AI fit and unfit groups

This study included 206 patients with an L858R mutation, and 244 patients with exon 19 deletion. There was no significant difference in the PFS between EGFR L858R and exon 19 deletion, and the median PFS in each group was 11.2 and 12.6 months, respectively (HR = 1.15, 95% CI: 0.93–1.41, *p* = 0.191; Figure 2C). In contrast, exon 19 deletion group had a significantly longer OS than the L858R mutation group; the median OS in patients with L858R and exon 19 deletion were 25.6 and 32.3 months, respectively (HR = 1.28, 95% CI: 1.03–1.60, *p* = 0.029; Figure 2D).

**FIGURE 3:**
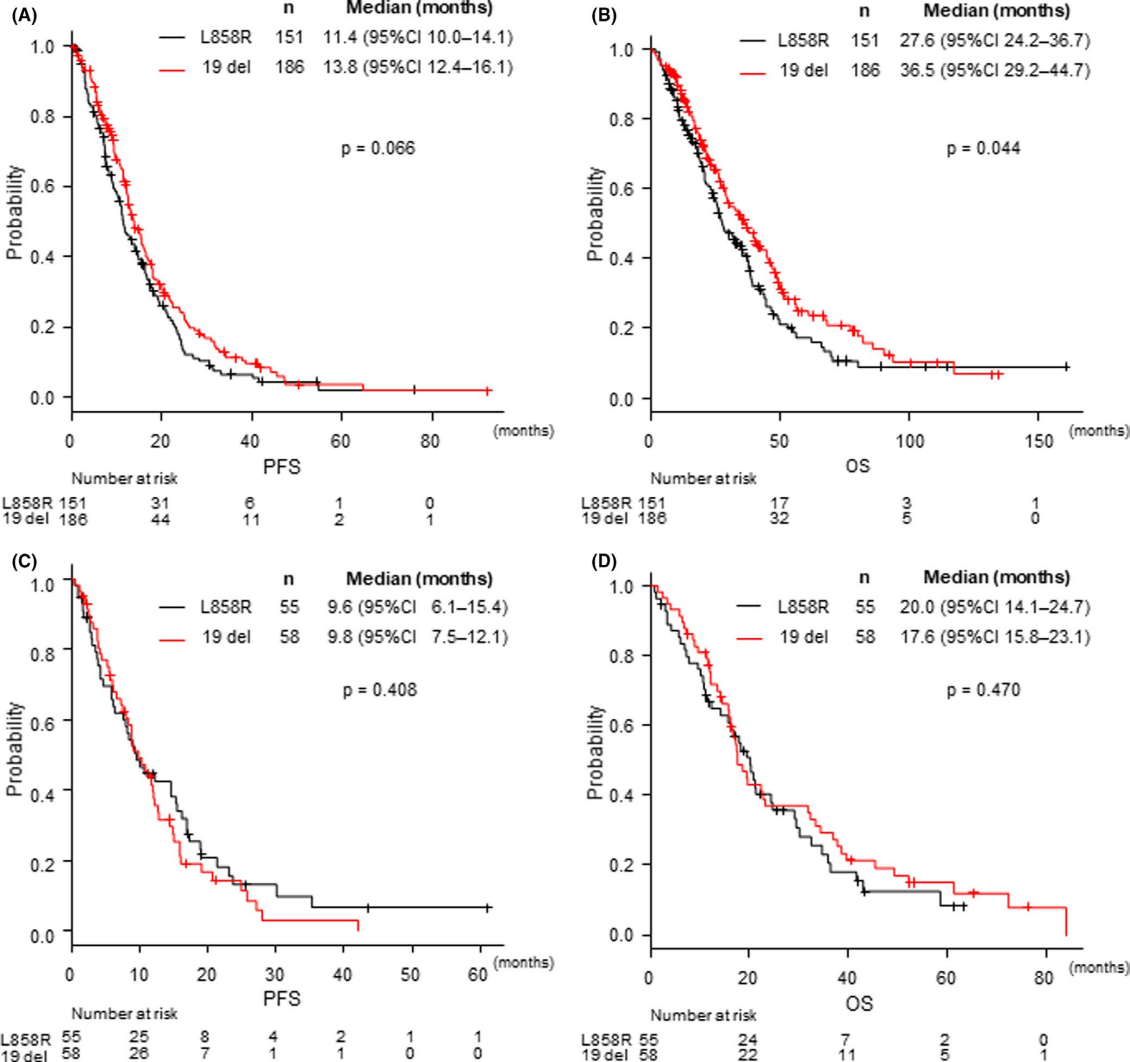
Kaplan–Meier curves for (A) progression‐free survival and (B) overall survival in the AI fit group, and (C) progression‐free survival and (D) overall survival in the AI unfit group. AI, angiogenesis inhibitor; CI, confidence interval; HR, hazard ratio; OS, overall survival; PFS, progression‐free survival

The AI fit group comprised 337 patients, including 44.8% (151/337) with an L858R mutation and 55.2% (186/337) with an exon 19 deletion. Median PFS in the AI fit subgroup was 11.4 months for L858R mutation and 13.8 months for exon 19 deletion; although it did not show a significant difference, 19 del resulted in a longer PFS (HR = 1.25, 95% CI: 0.98–1.59, *p* = 0.066; Figure 3A). In the AI fit group, multivariate analysis of PFS showed that relapsed stage, stage III (HR = 0.53, 95% CI: 0.40–0.70, *p* < 0.001), and PS 0–1 (HR = 0.62, 95% CI: 0.43–0.89, *p* = 0.009) were associated with a significantly longer PFS; however, the efficacy of EGFR‐TKI in the *EGFR* mutation subtypes was not significant (HR = 1.18, 95% CI: 0.93–1.50, *p* = 0.173; Table 3A). Moreover, OS was significantly shorter in patients with an L858R mutation (median OS = 27.6 months) than in those with an exon 19 deletion (36.5 months, HR = 1.31, 95% CI: 1.01–1.71, *p* = 0.044); however, multivariate analysis indicated that age (HR = 0.72, 95% CI: 0.54–0.97, *p* = 0.032), PS (HR = 0.62, 95% CI: 0.43–0.89, *p* = 0.009), and stage (HR = 0.64, 95% CI: 0.470.88, *p* = 0.006) were significant prognostic factors in the AI fit group, demonstrating that the *EGFR* mutation subtype (HR 1.27, 95% CI: 0.97–1.65, *p* = 0.082; Table 3B) was not an independent factor in the AI fit subgroup.

**TABLE 3:** Multivariate analysis in the AI fit subgroup

	*n*	%	Univariate		Multivariate	
			HR (95% CI)	*p* value	HR (95% CI)	*p* value
(A) PFS						
Age (<74/≧75)	241/96	71.5/28.5	1.15 (0.88–1.50)	0.296	1.15 (0.88–1.51)	0.297
Sex (male/female)	111/226	32.9/67.1	1.43 (1.11–1.84)	**0.005**	1.30 (0.93–1.83)	0.125
Stage (relapse, IIIB/IV)	94/243	27.9/72.1	0.52 (0.39–0.68)	**<0.001**	0.53 (0.40–0.70)	<**0.001**
PS (0,1/2)	290/47	86.1/13.9	0.65 (0.46–0.93)	**0.017**	0.62 (0.43–0.89)	**0.009**
Smoke (former, current/never)	190/147	56.4/43.6	1.38 (1.09–1.75)	**0.009**	1.14 (0.82–1.57)	0.432
*EGFR* (L858R/19 del)	151/186	44.8/55.2	1.25 (0.98–1.59)	0.066	1.18 (0.93–1.50)	0.173
TKI (first‐, second‐generation/osimertinib)	285/52	84.6/15.4	1.13 (0.74–1.72)	0.582	1.37 (0.89–2.10)	0.155
(B) OS						
Age (<74/≧75)	241/96	71.5/28.5	0.75 (0.56–1.00)	**0.048**	0.72 (0.54–0.97)	**0.032**
Sex (male/female)	111/226	32.9/67.1	1.54 (1.17–2.03)	**0.002**	1.37 (0.94–2.01)	0.102
Stage (relapse, IIIB/IV)	94/243	27.9/72.1	0.59 (0.43–0.81)	**0.001**	0.64 (0.47–0.88)	**0.006**
PS (0,1/2)	290/47	86.1/13.9	0.65 (0.46–0.93)	**0.017**	0.62 (0.43–0.89)	**0.009**
Smoke (former, current/never)	190/147	56.4/43.6	1.38 (1.09–1.75)	**0.009**	1.14 (0.82–1.57)	0.432
*EGFR* (L858R/19 del)	151/186	44.8/55.2	1.31 (1.01–1.71)	**0.044**	1.27 (0.97–1.65)	0.082
TKI (first‐, second‐generation/osimertinib)	285/52	84.6/15.4	1.09 (0.56–2.10)	0.798	1.19 (0.62–2.30)	0.600

Abbreviations: 19 del, exon 19 deletion; CI, confidence interval; *EGFR*, epidermal growth factor receptor; first‐, second‐generation, gefitinib, erlotinib, and afatinib; HR, hazard ratio; PFS, progression‐free survival; PS, performance status; TKI, tyrosine kinase inhibitor.

Bold values are *p* <0.05, which is statistically significant.

In the AI unfit group with 113 patients, both PFS and OS were equivalent between patients with an L858R mutation and those with an exon 19 deletion. The median PFS in L858R and del 19 group was 9.6 and 9.8 months, respectively (HR = 1.19, 95% CI: 0.79–1.78, *p* = 0.408), and the OS was 20.0 and 17.6 months, respectively (HR = 0.86, 95% CI: 0.56–1.30, *p* = 0.470; Figure 3C,D).

Multivariate analysis of the PFS in patients harboring an L858R mutation indicated that stage (HR = 0.47, 95% CI: 0.32–0.69, *p* < 0.001), PS (HR = 0.45, 95% CI: 0.30–0.68, *p* < 0.001), and a history of tumor exposure in the bronchus or producing bloody sputum (HR = 1.78, 95% CI: 1.09–2.90, *p* = 0.021) were related to a shorter PFS (Table 4A). In patients with an exon 19 deletion, only stage was an independent factor related to poor PFS (HR = 0.60, 95% CI: 0.41–0.86, *p* = 0.006; Table 4B).

**TABLE 4:** Univariate and multivariate analyses of PFS and OS in the *EGFR* L858R subtype and 19 del subtype

	PFS analysis				OS analysis			
	Univariate		Multivariate		Univariate		Multivariate	
	HR (95% CI)	*p* value	HR (95% CI)	*p* value	HR (95% CI)	*p* value	HR (95% CI)	*p* value
(A) L858R subtype								
Age (<74/≧75)	1.01 (0.72–1.41)	0.972	0.97 (0.68–1.37)	0.846	0.77 (0.54–1.10)	0.152	0.77 (0.53–1.13)	0.186
Sex (male/female)	1.22 (0.89–1.68)	0.219	1.23 (0.80–1.89)	0.355	1.39 (0.99–1.96)	0.056	1.48 (0.94–2.32)	0.087
Stage (relapse, IIIB/IV)	0.54 (0.39–0.76)	**<0.001**	0.47 (0.32–0.69)	**<0.001**	0.75 (0.52–1.09)	0.130	0.67 (0.44–0.99)	**0.046**
PS (0,1/2)	0.56 (0.38–0.82)	**0.003**	0.45 (0.30–0.68)	**<0.001**	0.54 (0.35–0.82)	**0.004**	0.45 (0.29–0.69)	**<0.001**
Smoke (former, current/never)	1.23 (0.91–1.67)	0.183	1.08 (0.72–1.63)	0.700	1.22 (0.88–1.70)	0.229	1.08 (0.70–1.66)	0.733
TKI (first‐, second‐gen/osimertinib)	1.29 (0.78–2.14)	0.323	1.38 (0.83–2.30)	0.218	1.27 (0.61–2.65)	0.526	1.19 (0.57–2.49)	0.646
History of CRT (yes/no)	0.77 (0.24–2.42)	0.654	1.65 (0.48–5.66)	0.430	1.01 (0.32–3.19)	0.984	1.64 (0.48–5.62)	0.433
MVI (yes/no)	1.18 (0.52–2.67)	0.691	0.86 (0.37–1.99)	0.730	1.79 (0.79–4.06)	0.167	1.22 (0.52–2.86)	0.647
CVD (yes/no)	0.92 (0.57–1.47)	0.721	1.27 (0.76–2.12)	0.352	1.56 (0.97–2.51)	0.065	1.69 (1.00–2.86)	0.052
Bloody sputum (yes/no)	1.62 (1.01–2.59)	**0.044**	1.78 (1.09–2.90)	**0.021**	1.55 (0.94–2.55)	**0.082**	1.94 (1.14–3.30)	**0.015**
(B) 19 del subtype								
Age (<74/≧75)	1.10 (0.81–1.49)	0.535	1.05 (0.77–1.43)	0.746	0.72 (0.52–1.00)	**0.047**	0.66 (0.47–0.93)	**0.018**
Sex (male/female)	1.45 (1.09–1.94)	**0.012**	1.22 (0.82–1.83)	0.326	1.64 (1.20–2.23)	**0.002**	1.47 (0.92–2.34)	0.102
Stage (relapse, IIIB/IV)	0.60 (0.43–0.83)	**0.002**	0.60 (0.41–0.86)	**0.006**	0.67 (0.47–0.95)	**0.025**	0.76 (0.51–1.12)	0.167
PS (0,1/2)	0.66 (0.43–1.00)	0.051	0.71 (0.45–1.13)	0.146	0.48 (0.31–0.75)	**0.001**	0.48 (0.30–0.75)	**0.002**
Smoke (former, current/never)	1.34 (1.01–1.78)	**0.040**	1.17 (0.79–1.73)	0.425	1.46 (1.07–1.98)	**0.017**	1.28 (0.80–2.04)	0.302
TKI (first‐, second‐generation/osimertinib)	1.44 (0.76–2.74)	0.262	1.69 (0.87–3.28)	0.124	1.17 (0.47–2.92)	0.734	1.31 (0.52–3.29)	0.571
History of CRT (yes/no)	1.35 (0.70–2.53)	0.375	1.68 (0.80–3.54)	0.170	1.08 (0.55–2.11)	0.834	1.14 (0.52–2.53)	0.738
MVI (yes/no)	1.96 (1.00–3.85)	0.051	1.58 (0.74–3.38)	0.241	1.69 (0.86–3.32)	0.126	1.51 (0.71–3.22)	0.282
CVD (yes/no)	1.94 (1.14–3.31)	**0.015**	1.47 (0.84–2.59)	0.181	1.86 (1.09–3.18)	**0.022**	1.10 (0.62–1.96)	0.731
Bloody sputum (yes/no)	1.63 (1.09–2.44)	**0.017**	1.18 (0.76–1.82)	0.467	1.64 (1.07–2.52)	**0.024**	1.42 (0.88–2.26)	0.148

Abbreviations: Bloody sputum, tumor exposure in the bronchus or producing bloody sputum; CI, confidence interval; CRT, chemoradiotherapy; CVD, cardiovascular disease; first‐, second‐gen, first‐ and second‐generation EGFR‐TKI; HR, hazard ratio; MVI, major vessel infiltration; PFS, progression‐free survival; PS, performance status; TKI, tyrosine kinase inhibitor.

Bold values are *p* <0.05, which is statistically significant.

## 4 DISCUSSION

In this study, we assessed the effect of AI eligibility in patients with an *EGFR*‐mutant NSCLC who had been treated with EGFR‐TKI monotherapy and evaluated the impact of AI eligibility for different *EGFR* mutation subtypes. As far as we know, this is the first study to assess the impact of AI eligibility between different *EGFR* subtypes.

Several factors, such as sex, history of smoking, *EGFR* subtypes (common/uncommon), ECOG PS, and clinical stages are related to *EGFR* mutation‐positive NSCLC prognosis.^12^, ^13^ Studies have shown that a selection bias in bevacizumab eligibility in patients with NSCLC treated with chemotherapy,^8^ and that eligibility for bevacizumab use is independently associated with OS in patients with *EGFR*‐mutant NSCLC.^14^ In accordance with these studies, we also observed significant differences in both PFS and OS of patients in AI fit and unfit groups, indicating that AI eligibility is related to prolonged PFS and OS in patients with NSCLC harboring an *EGFR* mutation. Therefore, we believe that there is a possibility that the results described in previous AI combination studies may have overestimated median PFS data or median OS data because of the selection bias due to AI eligibility. Our results also explain why erlotinib monotherapy in the NEJ026 study resulted in a relatively longer median PFS (13.3 months) and OS (46.2 months) than other erlotinib monotherapy studies (PFS = 10–13 months, OS = 23 months),^15^, ^16^ and even a longer OS than osimertinib monotherapy in the FLAURA study^17^ (38.6 months). Since several studies evaluating AI plus EGFR‐TKI combination therapies are ongoing, it is necessary to carefully interpret the PFS and OS data in these studies.

Previous phase III studies and meta‐analyses have shown that patients with an exon 19 deletion benefit more from EGFR‐TKI treatment than those with an L858R mutation. Accordingly, *EGFR* mutation subtype has been considered as an important stratified factor in recent clinical trials for patients with NSCLC harboring *EGFR* mutations.^1^, ^2^, ^3^, ^4^, ^5^ There are several rationales for this difference, including the prevalence of *de novo* T790M resistance mutation, tumor heterogeneity, difference in protein structure, and EGF‐induced tyrosine phosphorylation patterns.^18^, ^19^, ^20^ However, the multivariate analysis of the AI fit group showed that the *EGFR* subtype was not an independent factor for both PFS and OS. Our results imply that the prognosis of *EGFR* subtypes is comparable for patients that are potentially eligible for AI, irrespective of the AI used. Similar results can be observed in the NEJ 026 and RELAY studies, which demonstrated an equivalent median PFS in subgroups of *EGFR* mutations in the erlotinib monotherapy group.^6^, ^7^ In accordance with these results, it may be necessary to re‐evaluate the actual benefit of AIs and determine if the combination of AI with erlotinib could increase the rate of severe adverse events and mortality.^21^, ^22^

In this study, we also assessed the effects of all four factors of an AI fit condition. The multivariate analysis demonstrated that among the four factors, a history of tumor exposure in the bronchus or producing bloody sputum significantly affected both PFS and OS. This factor was likewise associated with PFS and OS in the L858R subgroup, but not in the 19 del subgroup. The presence of bloody sputum before treatment is reported to be a strong prognostic factor for advanced non‐squamous NSCLC.^8^ However, why the presence of bloody sputum and tumor exposure in the bronchus has a different impact on the L858R mutation and exon 19 deletion is yet to be elucidated. Some reports indicate that the location of the tumor, which could be related to the proportion of tumor exposure in the bronchus, is associated with PFS and OS in adenocarcinomas.^23^ Although our analysis showed that a central tumor causes a poor PFS and OS, the proportion of central or peripheral tumors did not differ between the L858R and exon 19 deletion groups. Thus, further considerations are required to clarify the mechanism underlying the difference in sensitivity between the *EGFR* subtypes.

The limitations of this study must be acknowledged. First, since this is a single‐center, retrospective study, there is a possibility of an unintentional selection bias. Further, unlike previous phase III studies and meta‐analyses of EGFR‐TKI treatment, the PFS in the ALL group was equivalent between 19 del and L858R subgroups, which might have affected the result for the AI fit and unfit groups.

## 5 CONCLUSION

Our study showed that the eligibility for AIs resulted in a longer PFS and OS and suggested that selection bias for AI eligibility could impact the selection of patients with NSCLC harboring an *EGFR* mutation. Moreover, the impact of AI eligibility may differ based on the *EGFR* subtype, which could, in turn, result in an unexpected selection bias in clinical trials and an overestimation of results. Since there is a possibility that such a selection bias affected previous trials evaluating the efficacy of EGFR‐TKI in combination with AI, the results of these trials should be carefully evaluated henceforth.

## CONFLICT OF INTEREST

H. K, T. K, A. N, M. Y, N. N, T. M, and E. M. has nothing to disclose. H. K. reports grants and personal fees from Chugai Pharmaceutical Co, Ltd, personal fees from Ono Pharmaceutical Co, Ltd, personal fees from Boeringer Ingelheim, personal fees from Eli Lilly K.K, personal fees from Kyowa Hakko Kirin Co. Ltd, personal fees from Bristol‐Myers Squibb, personal fees from MSD, grants and personal fees from Novartis Pharma K.K, grants and personal fees from Daiichi‐Sankyo Co. Ltd, grants and personal fees from AstraZeneca K.K, personal fees from Pfizer, and personal fees from Taiho Pharma outside the submitted work. N. M. reports personal fees from AstraZeneca KK, Pfizer Japan Inc, personal fees from Chugai Pharmaceutical Co. Ltd, grants and personal fees from Boehringer Ingelheim, personal fees from MSD K.K, personal fees from Taiho Pharmaceutical, and personal fees from Ono Pharmaceutical Co. Ltd outside the submitted work. H. K. reports personal fees from Eli Lilly K.K, personal fees from Taiho Pharmaceutical, and personal fees from AstraZeneca outside the submitted work. S. O. reports personal fees from Chugai Pharmaceutical Co. Ltd, personal fees from Ono Pharmaceutical Co. Ltd, personal fees from Taiho Pharmaceutical Co. Ltd, personal fees from Daiichi Sankyo Co. Ltd, personal fees from Amgen K.K., personal fees from AstraZeneca K.K, and personal fees from Novartis Pharma K.K outside the submitted work. K. W. reports grants and personal fees from Chugai Pharmaceutical CFo. Ltd, personal fees from Taiho Pharmaceutical, personal fees from Boehringer Ingelheim, personal fees from Eli Lilly K.K, personal fees from Ono Pharmaceutical, personal fees from MSD, grants and personal fees from Astrazeneca, grants from Novartis, and grants from Abbvie outside the sFDrubmitted work. T. N. reports grants from Ono Pharmaceutical Co. Ltd, grants from Pfizer US. Inc, and grants from Mochida Pharmaceutical Co. Ltd outside the submitted work. H. Murakami reports grants and personal fees from AstraZeneca, grants and personal fees from Chugai pharma, grants and personal fees from Takeda, grants and personal fees from Daiichi Sankyo, grants from Abbvie, grants from IQvia, personal fees from Ono Pharmaceutical, personal fees from Bristol‐Myers Squibb Japan, personal fees from MSD, personal fees from Pfizer, personal fees from Novartis, personal fees from Lilly Japan, and personal fees from Taiho Pharmaceutical outside the submitted work. T. T. reports grants and personal fees from AstraZeneca KK, Pfizer Japan Inc, grants and personal fees from Eli Lilly Japan K.K, grants and personal fees from Chugai Pharmaceutical Co. Ltd, grants and personal fees from Ono Pharmaceutical Co. Ltd, grants and personal fees from MSD K.K, grants and personal fees from Boehringer Ingelheim Japan Inc, grants and personal fees from Pfizer Japan, Inc, and personal fees from Roche Diagnostics K.K outside the submitted work.

## ETHICAL APPROVAL STATEMENT

This study was approved by the institutional review board of Shizuoka Cancer Center (IRB No. J2020‐170‐2020‐1), and performed under the ethical standards laid down in the 1964 Declaration of Helsinki and its later amendments. The need for informed consent was waived owing to the retrospective nature of the study.

## Data Availability

The data that support the findings of this study are available on request from the corresponding author. The data are not publicly available due to privacy or ethical restrictions.
